# Falling *Plasmodium knowlesi* Malaria Death Rate among Adults despite Rising Incidence, Sabah, Malaysia, 2010–2014

**DOI:** 10.3201/eid2201.151305

**Published:** 2016-01

**Authors:** Giri S. Rajahram, Bridget E. Barber, Timothy William, Matthew J. Grigg, Jayaram Menon, Tsin W. Yeo, Nicholas M. Anstey

**Affiliations:** Queen Elizabeth Hospital, Kota Kinabalu, Malaysia (G.S. Rajahram, T. William, J. Menon);; Infectious Diseases Society Sabah–Menzies School of Health Research Clinical Research Unit, Kota Kinabalu (B.E. Barber, T. William, M.J. Grigg, T.W. Yeo, N.M. Anstey);; Menzies School of Health Research, Darwin, Northern Territory, Australia (B.E. Barber, M.J. Grigg, T.W. Yeo, N.M. Anstey);; Charles Darwin University, Darwin (B.E. Barber, M.J. Grigg, T.W. Yeo, N.M. Anstey);; Jesselton Medical Centre, Kota Kinabalu (T. William);; Nanyang Technological University, Singapore (T.W. Yeo)

**Keywords:** malaria, severe malaria. Plasmodium knowlesi, Plasmodium vivax, Plasmodium falciparum, deaths, fatal malaria, death rate, fatality rate, artesunate, parasitemia, parasites, Malaysia, incidence

## Abstract

Deaths from *Plasmodium knowlesi* malaria have been linked to delayed parenteral treatment. In Malaysia, early intravenous artesunate is now recommended for all severe malaria cases. We describe *P. knowlesi* fatalities in Sabah, Malaysia, during 2012–2014 and report species-specific fatality rates based on 2010–2014 case notifications. Sixteen malaria-associated deaths (caused by PCR-confirmed *P. knowlesi* [7], *P. falciparum* [7], and *P. vivax* [1] and microscopy-diagnosed “*P. malariae*” [1]) were reported during 2012–2014. Six patients with severe *P. knowlesi* malaria received intravenous artesunate at hospital admission. For persons >15 years of age, overall fatality rates during 2010–2014 were 3.4, 4.2, and 1.0 deaths/1,000 *P. knowlesi*, *P. falciparum*, and *P. vivax* notifications, respectively; *P. knowlesi*–associated fatality rates fell from 9.2 to1.6 deaths/1,000 notifications. No *P. knowlesi*–associated deaths occurred among children, despite 373 notified cases. Although *P. knowlesi* malaria incidence is rising, the notification-fatality rate has decreased, likely due to improved use of intravenous artesunate.

## Abstract 2

The decreased notification-fatality rate is likely associated with improved use of intravenous artesunate for severe malaria.

*Plasmodium knowlesi* is the most common cause of malaria in East Malaysia, and the incidence of disease is increasing despite intensive control efforts that have substantially reduced the incidence of *P. falciparum* and *P. vivax* malaria in Malaysia (*1*–*3*). Although the greatest number of *P. knowlesi* cases has been reported in East Malaysia, the infection is also the predominant cause of malaria in Peninsular Malaysia (*4*) and is increasingly reported in other Southeast Asia countries and in travelers returning from these countries (*5*).

*P. knowlesi* infection can be associated with high parasitemia and is at least as likely as *P. falciparum* to cause severe malaria in adults (*6*). Age is strongly associated with parasitemia and, thus, a key risk factor for severe and fatal disease (*6*,*7*), neither of which has been reported in children with PCR-confirmed *P. knowlesi* malaria (*5*,*8*,*9*). In a tertiary referral hospital in Sabah, northeastern Malaysia, the rate of *P. knowlesi* malaria–associated deaths was low among persons >12 years of age who were promptly treated (including before hospital referral) with artesunate (*6*); however, *P. knowlesi* continues to cause fatal malaria among adults in Sabah (*10*,*11*). During 2010–2011, *P. knowlesi* was responsible for 6 of 14 fatal malaria cases in Sabah. Microscopy-based misdiagnosis of *P. knowlesi* malaria as the nearly identical, but more benign, *P. malariae* malaria was common, and fatal outcome was associated with delayed or lack of parenteral therapy: 2 of 6 patients with fatal *P. knowlesi* malaria received parenteral therapy (1 each with quinine and artesunate); the other 4 received chloroquine or sulfadoxine/pyrimethamine (*10*).

In the time since that study was conducted, recognition of *P. knowlesi* and its ability to cause severe disease has increased. The 2013 Management Guidelines of Malaria in Malaysia recommend that results for blood films with parasites resembling *P. malariae* be reported as *P. knowlesi/P. malariae* (*12*). The guidelines emphasize that PCR-confirmed *P. malariae* is rare in Sabah and that patients with a microscopy-based diagnosis of *P. malariae* infection should be assumed to have *P. knowlesi* malaria. Moreover, like recent World Health Organization (WHO) global guidelines (*13*–*15*), Malaysian guidelines now recommend intravenous artesunate for all patients with severe malaria caused by any *Plasmodium* spp. (*12*), and in western Sabah, the drug is being used earlier and more frequently for all malarial infections (*6*). In addition, oral artemisinin combination treatment is now recommended for uncomplicated *P. knowlesi* malaria (*12*).

We assessed clinical features and management of fatal *P. knowlesi* malaria cases in Sabah during 2012–2014. We also determined age-stratified death rates among patients with *Plasmodium* spp. malaria and assessed trends in fatality rates for *P. knowlesi* malaria during 2010–2014.

## Methods

In Sabah, which has an area of 73,600 km^2^ and population of 3.7 million (*16*), reporting of all malaria cases and associated deaths to the Sabah Department of Health (DoH) is mandatory; species are reported according to microscopy results. We obtained details of reported malaria-associated deaths during 2012–2014 from the Sabah DoH and reviewed district hospital case notes for clinical details. The study was approved by the ethics committees of the Malaysian Ministry of Health and Menzies School of Health Research.

We reviewed the Sabah DoH malaria notification database for the total number of microscopy-based *P. knowlesi*/*P. malariae, P. falciparum*, and *P. vivax* malaria case notifications during 2010–2014. These data were used to determine case-fatality rates (CFRs) among notified cases for each species (hereafter referred to as notified CFRs, defined as number of PCR-confirmed *P. knowlesi*, *P. falciparum*, and *P. vivax* malaria–associated deaths per 1,000 microscopy-based *P. malariae*/*P. knowlesi*, *P. falciparum*, and *P. vivax* malaria notifications). PCR-confirmed *P. malariae* infection is rare in Sabah, accounting for <1% of clinical samples diagnosed by microscopy as *P. malariae* or *P. knowlesi* (*1*); thus, most notifications of *P. malariae/P. knowlesi* can be assumed to be *P. knowlesi*. During 2010–2014 in Sabah, only 1 fatal malaria case, a microscopy-diagnosed *P. malariae* infection, lacked PCR confirmation; an adjusted CFR was calculated following inclusion of this case.

## Results

Sixteen malaria-associated deaths were reported in Sabah during 2012–2014, of which 15 were confirmed by PCR to be caused by *P. knowlesi* (7 cases; cases 1–7, Technical Appendix); *P. falciparum* (7 cases); or *P. vivax* (1 case). Details of 1 *P. knowlesi* case (case 2) were previously reported (*11*). The remaining fatal case was microscopy diagnosed (without PCR confirmation) as *P. malariae* infection (Technical Appendix).

### Fatal PCR-Confirmed *P. knowlesi* Malaria

All 7 fatal *P. knowlesi* malaria cases occurred in adults (median age 61 [range 31–73] years); 4 were women. Six of these cases had been misdiagnosed by microscopy as *P. malariae* (4), *P. falciparum* (1), or *P. vivax* (1) infections. Severe malaria was recognized in 5 patients when they sought medical care; all received intravenous artesunate within 90 (median 30) minutes of diagnosis. Severity criteria at admission for these patients were jaundice (4 patients), acute kidney injury (3), metabolic acidosis (4), hyperparasitemia (2), respiratory distress (2), and coma (1) (Technical Appendix Tables 1, 2). Shock and respiratory distress developed in all patients before death. All patients were intubated and ventilated; 2 received hemodialysis. Death occurred within 5–117 (median 41) hours of admission.

Two patients with fatal *P. knowlesi* malaria were not recognized to have severe malaria at admission; they received oral antimalarial treatment. One of these patients (case-patient 7, Technical Appendix Table 1) had a blood film result reported as 22,666 *P. malariae* parasites/μL and was given oral artesunate/mefloquine for apparent uncomplicated malaria. Her creatinine level was 124 (reference 63–133) μmol/L; bilirubin, lactate, and bicarbonate results were not available. Within 12 hours, she became hypotensive and tachypneic; chest radiographs showed diffuse opacities in both lung fields. She was intubated and started on intravenous artesunate but died within 23 hours of admission. Subsequent reexamination of her initial blood slide showed 263,772 parasites/μL. The other patient (case 5, Technical Appendix Table 1) was also thought to have uncomplicated malaria; her blood film result was reported as 9,866 *P. vivax* parasites/μL, her bilirubin level was 46 (reference <17) μmol/L, and her creatinine level was 143 μmol/L. She was treated with 1 dose of intravenous artesunate followed by chloroquine and primaquine. Blood film results the next day indicated *P. knowlesi* infection with 20,000 parasites/μL. Acute respiratory distress syndrome (ARDS) and metabolic acidosis developed, and the patient died on day 3, despite recommencement of intravenous artesunate. Postmortem reexamination of her initial blood slide showed 55,111 *P. knowlesi* parasites/μL.

One patient, a 56-year-old man, was comatose, a condition not previously reported in *P. knowlesi* malaria. He was unresponsive when brought into a health clinic by relatives, who reported a 1-day history of weakness and drowsiness and a 3-day history of fever, chills, arthralgia, and myalgia. At hospital referral, his blood pressure was 85/60 mm Hg, pulse rate 150 beats/min, and oxygen saturation 81% on room air. He had a Glasgow Coma Scale score of 6/15. Pupils were reactive but asymmetric (right 2 mm, left 4 mm). Meningism was not present, and neurologic examination showed normal tone, symmetrically reduced reflexes, and downgoing plantar reflexes. Blood investigations showed 6,471 *P. knowlesi* parasites/μL and metabolic acidosis. Computed tomography brain scan and lumbar puncture were not performed. The patient was intubated and begun on intravenous artesunate and ceftriaxone but died 41 hours after admission. Blood cultures for bacterial infections were negative.

### Fatal PCR-Confirmed *P. vivax* Malaria

One person, a 53-year-old man, died from *P. vivax* malaria. At admission, he had a 7-day history of fever, rigors, myalgia, nonproductive cough, and abdominal pain. Physical examination results were unremarkable. Thrombocytopenia was present, and his bilirubin level was 21.5 μmol/L; creatinine and hemoglobin levels were normal. A blood film result was reported as 2,090 *P. vivax* parasites/μL; oral chloroquine and primaquine treatment were begun. The next day, the parasite count was 890 parasites/μL of blood, but the patient became hypotensive, and ARDS developed; a postintubation chest radiograph showed bilateral opacities. Intravenous artesunate and antibiotic drugs were initiated, and hemodialysis was performed for acute kidney injury (creatinine level 316 μmol/L), but the patient died 4 days after admission. Blood cultures for bacterial infections were negative.

### Fatal PCR-Confirmed *P. falciparum* Malaria

Of the 7 *P. falciparum* malaria–associated deaths, 2 (29%) occurred in children (a boy and a girl 2–3 years of age, both Filipino) and 5 (71%) occurred in adults (3 men and 2 women 31–80 years of age; 2 Filipino, 2 Malaysian, and 1 Indonesian). At the initial examination, all patients met WHO criteria for severe malaria: jaundice (5 patients), cerebral malaria (4 patients), renal failure (3 patients), respiratory distress (3 patients), and anemia (2 patients). Within 2 hours of malaria diagnosis, 1 patient was given oral artesunate/mefloquine and all others were given intravenous artesunate. All patients were intubated and ventilated, 6 received inotropes, and dialysis was performed on 3. All patients died within 2–9 days of admission. Blood cultures for 1 child and 1 adult were positive for *Klebsiella pneumoniae* and coagulase-negative staphylococci, respectively; the latter was thought to represent contamination.

### Review of 2010–2014 Malaria Notification Data

The overall notified CFR for *P. knowlesi* malaria was 3.08 deaths/1,000 cases, compared with 4.83 and 0.87 deaths/1,000 cases of *P. falciparum* and *P. vivax* malaria, respectively (Table). Among adults (persons >15 years of age), notified CFRs were 3.37, 4.17, and 1.02 deaths/1,000 cases for *P. knowlesi*, *P. falciparum*, and *P. vivax* malaria, respectively. Despite 373 notifications of *P. knowlesi* malaria and 611 notifications of *P. vivax* malaria among children (persons <15 years of age) during 2010–2014, no deaths from either species were reported. However, children with *P. falciparum* malaria had a notified CFR of 6.7 deaths/1,000 cases. Notified CFRs among adults with *P. knowlesi* malaria declined from 9.2 to 1.6 deaths/1,000 notifications in 2010 and 2014, respectively (χ^2^ test for trend, p = 0.11).

**Table:** CFRs among persons with notified cases of *Plasmodium* spp. malaria, Sabah, Malaysia, 2010–2014*

Age group, year	*P. knowlesi*†				*P. falciparum*					*P. vivax*		
	No. notifications	No. deaths	Notified CFR‡		No. notifications	No. deaths	Notified CFR			No. notifications	No. deaths	Notified CFR
Persons >15 y of age												
2010	327	3	9.17		736	2	2.72			720	0	0.00
2011	608	3	4.93		467	2	4.28			479	1	2.09
2012	744	4§	5.38		543	2	3.68			390	0	
2013	927	1	1.08		239	1	4.18			211	1	4.74
2014	1,246	2	1.61		173	2	11.56			165	0	
Total	3,852	13	3.37		2,158	9	4.17			1,965	2	1.02
Persons <15 y of age												
2010	57	0	–		355	2	5.63			262	0	–
2011	95	0	–		138	1	7.25			149	0	–
2012	73	0	–		171	2	11.70			88	0	–
2013	69	0	–		58	0	–			52	0	–
2014	79	0	–		21	0	–			60	0	–
Total	373	0	–		743	5		6.73		611	0	–

*Notified CFR, case-fatality rate determined on the basis of the no. of PCR-confirmed malaria-associated deaths/1,000 notifications of microscopy-based malaria cases. †Includes all cases notified as *P. knowlesi* or *P. malariae*. PCR-confirmed *P. malariae* infection is rare in Sabah, accounting for <1% of clinical samples diagnosed by microscopy as *P. malariae* or *P. knowlesi* (*1*); thus, most notifications of *P. malariae/P. knowlesi* can be assumed to be *P. knowlesi*. ‡p = 0.110 (χ^2^ test for trend) for reduction in *P. knowlesi* fatality rate among adults during 2010–2014. §Excludes 1 fatal case of microscopy-diagnosed *P. malariae*. The notified CFR in 2012 with this case included as a *P. knowlesi*–associated death was 6.72 deaths/1,000 notifications, and the overall notified CFR for *P. malariae/P. knowlesi* in adults was 3.63 deaths/1,000 notifications. The p value for the test for trend is unchanged at 0.11

During 2010–2014, female patients accounted for only 783 (19%) of the 4,217 *P. malariae/P. knowlesi* notifications, but they accounted for 6 (46%) of the 13 fatal *P. knowlesi* malaria cases. Thus, the notified CFR for *P. knowlesi* malaria was 7.66 deaths/1,000 cases for female patients, compared with 2.04 deaths/1,000 cases for male patients (Fisher exact test, p = 0.021). However, this difference was not significant in a multivariate logistic regression model adjusting for age (odds ratio 2.60, p = 0.095). For *P. falciparum* and *P. vivax* malaria, no difference was seen in the number of notified CFRs for male and female patients.

## Discussion

Despite ongoing microscopy-based misdiagnoses of *P. knowlesi* infections, management of severe malaria in Sabah appears to have improved; all patients recognized to have severe *P. knowlesi* malaria on admission received intravenous artesunate as initial therapy. Although our findings clearly demonstrate the ability of *P. knowlesi* to cause fatal malaria despite optimal therapy, *P. knowlesi* notified CFRs in Sabah have fallen over the past 5 years in association with the increased early use of artesunate documented in this and other reports (*6*). Death from *P. knowlesi* malaria remains unreported in children, and all but 1 of the *P. knowlesi*–associated deaths in this series occurred in adults >50 years of age.

The absence of *P. knowlesi*–associated deaths among children, despite 373 *P. malariae*/*P. knowlesi* notified cases in this age group during 2010–2014, contrasts with the well-recognized risk for childhood deaths from *P. falciparum* malaria (*17*) and extends the lack of previous reports of either severe or fatal outcomes in children with *P. knowlesi* malaria (*5*,*8*,*9*). Furthermore, the large number of notified cases in children in this series suggests that the lack of *P. knowlesi*–associated deaths among children may not be due solely to the relative underrepresentation of children in previous series of *P. knowlesi* malaria (*6*,*7*,*18*,*19*). A lower risk for severe and fatal *P. knowlesi* malaria in children may be due to the previously documented strong association between age and parasitemia (*6*); the level of parasitemia in children is generally insufficient to cause severe and fatal disease (*20*). In addition, younger age may be associated with physiologic protection from severe and fatal *P. knowlesi* malaria, as suggested by previous findings of a lower risk of severe malaria after primary exposure to *P. falciparum* in nonimmune children compared with nonimmune adults (*21*).

Clinical and demographic characteristics for adult *P. knowlesi* malaria patients in this study were consistent with those in previous reports (*1*,*6*,*7*,*18*). Patients had a median age of 61 years. Because of the strong correlation between age and parasitemia, older patients are known to be at increased risk for severe *P. knowlesi* malaria (*6*). Of the 7 patients who died, 4 were female; thus the notified CFR was significantly higher among female than male patients. Although this discrepancy appears to be primarily due to the older age of female patients with *P. knowlesi* malaria (*1*), a trend toward increased notified CFRs for female *P. knowlesi* patients remained even after adjusting for age. This finding is consistent with the increased risk for severe *P. knowlesi* malaria found for female patients in some (*7*,*18*), but not all (*6*), previous studies. Larger studies are needed to clarify the association between sex and risk for severe *P. knowlesi* malaria.

The complications experienced by the *P. knowlesi* malaria patients in this study were generally consistent with those in other reports; hyperparasitemia, respiratory distress, shock, jaundice, and acute kidney injury were common in this and previous reports (*6*,*7*,*18*,*22*–*24*). Metabolic acidosis occurred in 5 patients in this series. This complication of severe *P. knowlesi* malaria was uncommon in a previous tertiary referral hospital study that involved early, including prereferral, use of artesunate and in which no deaths occurred (*6*). However, metabolic acidosis has been reported in most fatal *P. knowlesi* malaria cases (*7*,*10*,*11*,*18*,*25*), and, as with *P. falciparum* malaria, is likely a late complication signifying poor outcome. Acute lung injury was present at admission in 2 patients and developed after treatment initiation in all remaining *P. knowlesi* patients; this finding is consistent with a posttreatment inflammatory response, as previously postulated (*6*).

Coma has not previously been reported in *P. knowlesi* malaria. Although decreased conscious state occurred in 1 patient in this study, blood cultures, lumber puncture, and computed tomography brain scan were not performed, and pupillary asymmetry, reported in this patient, is unusual in coma due to *P. falciparum* malaria. Therefore, while decreased consciousness directly associated with *P. knowlesi* remains possible, alternative causes are plausible.

Microscopy-based misdiagnosis of *P. knowlesi* infection occurred in 6 of 7 cases. Four of the 6 case-patients had misdiagnoses of *P. malariae* infection; all had high parasitemia (2 had >100,000 parasites/µL of blood), which is inconsistent with a diagnosis of *P. malariae* infection but highly suggestive of *P. knowlesi* infection. Malaysia’s malaria guidelines recommend that blood films with parasites resembling *P. malariae* be reported as *P. knowlesi/P. malariae* (*12*); however, high parasitemia, particularly in the context of a very low statewide prevalence of *P. malariae* (*1*), makes *P. malariae* infection unlikely. The frequent microscopy-based misdiagnosis of *P. knowlesi* malaria in this and other reports (*26*) in Sabah highlights the need for alternative rapid diagnostic methods.

Despite the frequent misdiagnoses in this series, all patients with fatal *P. knowlesi* infection who were recognized as having severe malaria at admission were appropriately treated with early intravenous artesunate. In contrast, in our 2010–2011 review of malaria deaths (*10*), only 2 of 5 patients with severe *P. knowlesi* malaria received parenteral treatment; the other 3, who had misdiagnoses of *P. malariae* or *P. vivax* malaria, received oral chloroquine or sulfadoxine/pyramethamine. In the current study, 2 patients were thought to have uncomplicated malaria and received oral therapy (case-patient 5 was given chloroquine after 1 dose of intravenous artesunate; case-patient 7 was given artesunate/mefloquine). Postmortem reexamination of these patients’ initial blood films showed a parasite count substantially higher than initially reported (55,111 parasites/μL vs. 9,866 parasites/μL for case-patient 5; 263,772 parasites/μL vs. 22,666 parasites/μL for case-patient 7). Parasitemia has been shown to be a major risk factor for severe *P. knowlesi* malaria: in a recent prospective study, severity criteria were present in >50% of patients with >20,000 parasites/μL of blood and >80% of patients with >100,000 parasites/μL of blood (*6*). WHO guidelines now recommend that intravenous artesunate be used for all patients with *P. knowlesi* malaria and >100,000 parasites/μL of blood or, if testing for laboratory criteria for severe malaria is not available, >20,000 parasites/μL blood (*14*,*15*). The failure of oral therapy in case-patient 7 (initial blood slide reported as 22,666 parasites/μL; bilirubin not available) highlights the value of this recommendation. Moreover, these 2 cases demonstrate that parasitemia must be accurately quantified in patients with *P. knowlesi* malaria.

The use of chloroquine in case-patient 5 may have contributed to the poor outcome. Compared with artesunate/mefloquine, chloroquine has been associated with reduced parasite clearance time in *P. knowlesi* malaria (*27*,*28*) and is no longer recommended as first-line treatment for *P. knowlesi* malaria in Malaysia (*12*). In Sabah, parasite clearance time for *P. vivax* malaria treated with chloroquine is reduced compared with that for cases treated with artesunate/mefloquine, and treatment failures are common (*29*). Moreover, *P. knowlesi* and *P. vivax* are frequently confused in microscopy examination (*26*); hence, a unified treatment approach should be considered in Sabah, using artemisinin for all malaria cases.

Although this case series highlights the ability of *P. knowlesi* malaria to cause fatal disease in adults even after prompt administration of intravenous artesunate, it must be noted that the number of deaths has not increased over recent years, despite a rise in *P. knowlesi* malaria notifications from 384 in 2010 to 1,325 in 2014. Thus, the adult notified CFR has declined from 9.2 deaths/1,000 notifications in 2010 to 1.6 deaths/1,000 notifications in 2014. This improvement likely resulted from increased recognition of the ability of *P. knowlesi* to cause severe disease and to increased use of intravenous artesunate. Although intravenous artesunate has been recommended in Sabah since December 2008, intravenous quinine was still in use until at least 2010 (*10*). In a retrospective study of severe *P. knowlesi* malaria at a tertiary referral hospital in Sabah during 2007–2009, a total of 5 (31%) of 16 patients treated with intravenous quinine died. In contrast, at the same hospital during 2010–2011, none of the severe *P. knowlesi* malaria patients treated with early intravenous artesunate died (*6*). In addition, in Sabah, the increasing use of artemisinin combination treatment instead of chloroquine for uncomplicated *P. knowlesi* malaria (*6*) may also have contributed to the decline in notified CFRs, particularly for cases in which severe disease is unrecognized.

We also reported a case of fatal *P. vivax* malaria with ARDS, an increasingly well-recognized complication of *P. vivax* malaria that has resulted in fatalities (*30*–*34*). The pathophysiologic mechanism likely involves soluble mediators and endothelial damage, exacerbated by shock and leading to diffuse damage to alveolar membranes (*30*). As with the case in our study, most ARDS cases occur after treatment initiation (*32*,*35*), possibly resulting from an exacerbated inflammatory response to parasite killing. Most of the initial cases of *P. vivax*–associated ARDS were in returned travelers with single organ dysfunction and nonfatal outcome; however, more recent series from countries where *P. vivax* is endemic have reported ARDS cases with multiorgan dysfunction and considerable mortality (*35*,*36*).

This study had several limitations. First, the retrospective design of the case series resulted in unavoidably incomplete laboratory and clinical data. In particular, alternative diagnoses cannot be excluded in the case of possible *P. knowlesi*–associated coma. Second, our calculation of the microscopy-based notified CFR represents only an estimate of the true *P. knowlesi–*associated CFR. The accuracy of this estimate will depend on the accuracy of microscopy-based identification of all *Plasmodium* species, the notification rate of malaria cases, and the proportion of persons with malaria who seek care at a health clinic. We do not have data on the proportion of malaria cases in Sabah that are notified; it is probable, however, that some are not notified, so the notified CFR likely overestimates the true CFR. In addition, we cannot exclude the possibility that the reduction in the *P. knowlesi*–associated notified CFRs during 2010–2014 is due to an increase in the proportion of malaria cases that are notified. However, notification of malaria cases in Sabah has been mandatory since 1992, and there is no reason to suspect that the notification rate would have changed substantially since 2010. It is similarly unlikely that the proportion of *P. knowlesi* malaria cases diagnosed as *P. falciparum* malaria, and vice versa, changed sufficiently over the 5-year period to account for the observed decline in notified CFRs (*1*). Nonetheless, larger prospective studies involving molecular diagnostic methods are needed to obtain a more accurate assessment of the true *P. knowlesi* malaria CFR, including changes over time. Although we report notified CFRs for *P. knowlesi*, *P. falciparum*, and *P. vivax* malaria, these data may not reflect the relative virulence of each species. In this series, non-Malaysian citizens accounted for a higher proportion (5/7) of patients with fatal *P. falciparum* malaria than fatal *P. knowlesi* malaria, and it is possible that a delay in seeking care at a healthcare facility may be a confounding factor in comparing CFRs for malaria caused by these *Plasmodium* spp.

In conclusion, our findings show that despite increasing notifications of *P. knowlesi* malaria cases in Sabah, the number of fatal cases has not increased. The reduction in notified CFRs may be associated with the increased recognition of the ability of *P. knowlesi* to cause severe and fatal malaria and improved use of intravenous artesunate for severe malaria caused by any *Plasmodium* spp, as per recent policy changes (*6*,*12*). Nonetheless, this study demonstrates the ability of *P. knowlesi* to cause fatal malarial disease in adults, despite optimal therapy, and that *P. knowlesi* remains the most common cause of fatal malaria in adults in Sabah. In contrast, the study shows a notable absence of deaths among children with *P. knowlesi* malaria.

Technical Appendix Detailed descriptions of the 7 fatal cases of *Plasmodium knowlesi* malaria and the fatal *P. vivax* and microscopy-diagnosed *P. malariae* cases identified in Sabah, Malaysia, 2010–2014.

## References

[1] William T, Jelip J, Menon J, Anderios F, Mohammad R, Awang Mohammad TA, Changing epidemiology of malaria in Sabah, Malaysia: increasing incidence of *Plasmodium knowlesi.* Malar J. 2014;13:390. 10.1186/1475-2875-13-390 25272973 PMC4195888

[2] William T, Rahman HA, Jelip J, Ibrahim MY, Menon J, Grigg MJ, Increasing incidence of *Plasmodium knowlesi* malaria following control of *P. falciparum* and *P. vivax* malaria in Sabah, Malaysia. PLoS Negl Trop Dis. 2013;7:e2026. 10.1371/journal.pntd.0002026 23359830 PMC3554533

[3] Sarawak State Health Department. Sarawak EPID News [cited 2015 Jun 6]. http://jknsarawak.moh.gov.my/en/modules/wfdownloads/viewcat.php?cid=31

[4] Yusof R, Lau YL, Mahmud R, Fong MY, Jelip J, Ngian H, High proportion of knowlesi malaria in recent malaria cases in Malaysia. Malar J. 2014;13:168. 10.1186/1475-2875-13-168 24886266 PMC4016780

[5] Singh B, Daneshvar C. Human infections and detection of *Plasmodium knowlesi.* Clin Microbiol Rev. 2013;26:165–84. 10.1128/CMR.00079-12 23554413 PMC3623376

[6] Barber BE, William T, Grigg MJ, Menon J, Auburn S, Marfurt J, A prospective comparative study of knowlesi, falciparum and vivax malaria in Sabah, Malaysia: high proportion with severe disease from *Plasmodium knowlesi* and *P. vivax* but no mortality with early referral and artesunate therapy. Clin Infect Dis. 2013;56:383–97. 10.1093/cid/cis902 23087389

[7] William T, Menon J, Rajahram G, Chan L, Ma G, Donaldson S, Severe *Plasmodium knowlesi* malaria in a tertiary hospital, Sabah, Malaysia. Emerg Infect Dis. 2011;17:1248–55. 10.3201/eid1707.101017 21762579 PMC3381373

[8] Barber BE, William T, Jikal M, Jilip J, Dhararaj P, Menon J, *Plasmodium knowlesi* malaria in children. Emerg Infect Dis. 2011;17:814–20. 10.3201/eid1705.101489 21529389 PMC3321776

[9] Barber BE, William T, Dhararaj P, Anderios F, Grigg MJ, Yeo TW, Epidemiology of *Plasmodium knowlesi* malaria in northeast Sabah, Malaysia: family clusters and wide age distribution. Malar J. 2012;11:401. 10.1186/1475-2875-11-401 23216947 PMC3528466

[10] Rajahram GS, Barber BE, William T, Menon J, Anstey NM, Yeo TW. Deaths due to *Plasmodium knowlesi* malaria in Sabah, Malaysia: association with reporting as *P. malariae* and delayed parenteral artesunate. Malar J. 2012;11:284. 10.1186/1475-2875-11-284 22905799 PMC3472242

[11] Rajahram GS, Barber BE, Yeo TW, Tan WM, William T. Case report: fatal *Plasmodium knowlesi* malaria following an atypical clinical presentation and delayed diagnosis. Med J Malaysia. 2013;68:71–2 . 23466773

[12] Ministry of Health Malaysia. Management guidelines of malaria in Malaysia. 2014 [cited 2015 Aug 15]. http://www.moh.gov.my/english.php/pages/view/118

[13] World Health Organization. Guidelines for the treatment of malaria. 3rd edition. Geneva: The Organization; 2015.

[14] World Health Organization. Management of severe malaria: a practical handbook. 3rd edition. Geneva: The Organization; 2013.

[15] World Health Organization. Severe malaria. Trop Med Int Health. 2014;19(Suppl 1):7–131. 10.1111/tmi.12313_2 25214480

[16] Department of Statistics Malaysia. Statistics. Sabah [cited 2015 Jun 6]. https://www.statistics.gov.my/index.php?r=column/cone&menu_id=dTZ0K2o4YXgrSDRtaEJyVmZ1R2h5dz09

[17] Dondorp AM, Fanello CI, Hendriksen IC, Gomes E, Seni A, Chhaganlal KD, Artesunate versus quinine in the treatment of severe falciparum malaria in African children (AQUAMAT): an open-label, randomised trial. Lancet. 2010;376:1647–57. 10.1016/S0140-6736(10)61924-1 21062666 PMC3033534

[18] Daneshvar C, Davis TM, Cox-Singh J, Rafa’ee M, Zakaria S, Divis P, Clinical and laboratory features of human *Plasmodium knowlesi* infection. Clin Infect Dis. 2009;49:852–60. 10.1086/605439 19635025 PMC2843824

[19] Willmann M, Ahmed A, Siner A, Wong I, Woon L, Singh B, Laboratory markers of disease severity in *Plasmodium knowlesi* infection: a case control study. Malar J. 2012;11:363. 10.1186/1475-2875-11-363 23110615 PMC3533741

[20] Grigg MJ, William T, Menon J, Barber B, Wilkes CS, Price R, Disease burden from *P. vivax* and *P. knowlesi* malaria in children in Sabah, Malaysia. In: Abstracts of the 5th International Conference of Research on *Plasmodium vivax* Malaria; Jimbaran, Bali, Indonesia; 2015 May 19–22. Jakarta: Eijkman–Oxford Clinical Research Unit; 2015.

[21] Baird JK, Masbar S, Basri H, Tirtokusumo S, Subianto B, Hoffman SL. Age-dependent susceptibility to severe disease with primary exposure to *Plasmodium falciparum.* J Infect Dis. 1998;178:592–5. 10.1086/517482 9697752

[22] Cox-Singh J, Davis TM, Lee KS, Shamsul SS, Matusop A, Ratnam S, *Plasmodium knowlesi* malaria in humans is widely distributed and potentially life threatening. Clin Infect Dis. 2008;46:165–71. 10.1086/524888 18171245 PMC2533694

[23] Ahmed MA, Cox‐Singh J. *Plasmodium knowlesi*—an emerging pathogen. ISBT Science Series. 2015;10(Suppl 1):134–40. 10.1111/voxs.12115 PMC4440384 26029250

[24] Lee CE, Adeeba K, Freigang G. Human *Plasmodium knowlesi* infections in Klang Valley, Peninsular Malaysia: a case series. Med J Malaysia. 2010;65:63–5 . 21265252

[25] Cox-Singh J, Hiu J, Lucas SB, Divis PC, Zulkarnaen M, Chandran P, Severe malaria—a case of fatal *Plasmodium knowlesi* infection with post-mortem findings. Malar J. 2010;9:10. 10.1186/1475-2875-9-10 20064229 PMC2818646

[26] Barber BE, William T, Grigg MJ, Yeo TW, Anstey NM. Limitations of microscopy to differentiate *Plasmodium* species in a region co-endemic for *Plasmodium falciparum, Plasmodium vivax* and *Plasmodium knowlesi.* Malar J. 2013;12:8. 10.1186/1475-2875-12-8 23294844 PMC3544591

[27] Grigg MJ, William T, Dhanaraj P, Menon J, Barber BE, von Seidlein L, A study protocol for a randomised open-label clinical trial of artesunate–mefloquine versus chloroquine in patients with non-severe *Plasmodium knowlesi* malaria in Sabah, Malaysia (ACT KNOW trial). BMJ Open. 2014;4:e006005. 10.1136/bmjopen-2014-006005 PMC4139630 25138814

[28] Grigg MJ, William T, Dhanaraj P, Menon J, Barber B, von Seidlein L, A randomized open-label clinical trial of artesunate–mefloquine versus chloroquine in patients with non-severe *Plasmodium knowlesi* malaria in Sabah, Malaysia (ACT KNOW trial). Lancet Infect Dis. 2015. In press. 10.1016/S1473-3099(15)00415-6 PMC4139630 25138814

[29] Grigg MJ, William T, Menon J, Dhanaraj P, Barber B, Wilkes CS, A randomized open-label clinical trial of artesunate–mefloquine versus chloroquine in patients with non-severe *Plasmodium vivax* malaria in Sabah, Malaysia. In: Abstracts of the 5th International Conference of Research on *Plasmodium vivax* Malaria; Jimbaran, Bali, Indonesia; 2015 May 19–22. Jakarta: Eijkman–Oxford Clinical Research Unit; 2015.

[30] Valecha N, Pinto RGW, Turner GDH, Kumar A, Rodrigues S, Dubhashi NG, Histopathology of fatal respiratory distress caused by *Plasmodium vivax* malaria. Am J Trop Med Hyg. 2009;81:758–62. 10.4269/ajtmh.2009.09-0348 19861606

[31] Lacerda MVG, Fragoso SCP, Alecrim MGC, Alexandre MAA, Magalhães BML, Siqueira AM, Postmortem characterization of patients with clinical diagnosis of *Plasmodium vivax* malaria: to what extent does this parasite kill? Clin Infect Dis. 2012;55:e67–74. 10.1093/cid/cis615 22772803

[32] Anstey NM, Douglas NM, Poespoprodjo JR, Price R. *Plasmodium vivax*: clinical spectrum, risk factors and pathogenesis. Adv Parasitol. 2012;80:151–201. 10.1016/B978-0-12-397900-1.00003-7 23199488

[33] Douglas NM, Pontororing G, Lampah D, Yeo T, Kenangalem E, Poespoprodjo J, Mortality attributable to *Plasmodium vivax* malaria: a clinical audit from Papua, Indonesia. BMC Med. 2014;12:217. 10.1186/s12916-014-0217-z 25406857 PMC4264333

[34] McGready R, Wongsaen K, Chu C, Tun N, Chotivanich K, White N, Uncomplicated *Plasmodium vivax* malaria in pregnancy associated with mortality from acute respiratory distress syndrome. Malar J. 2014;13:191. 10.1186/1475-2875-13-191 24886559 PMC4046059

[35] Anstey NM, Russell B, Yeo TW, Price RN. The pathophysiology of vivax malaria. Trends Parasitol. 2009;25:220–7. 10.1016/j.pt.2009.02.003 19349210

[36] Siqueira AM, Lacerda MV, Magalhães BM, Mourão MP, Melo GC, Alexandre MA, Characterization of *Plasmodium vivax*–associated admissions to reference hospitals in Brazil and India. BMC Med. 2015;13:57. 10.1186/s12916-015-0302-y 25889040 PMC4404636

