# Supplementary material for: Falling Plasmodium knowlesi Malaria Death Rate among Adults despite Rising Incidence, Sabah, Malaysia, 2010–2014
Source: Emerg Infect Dis. 2016 Jan;22(1):41–8. doi: 10.3201/eid2201.151305 (PMC4696710; doi:10.3201/eid2201.151305)
Supplement: Technical Appendix — Detailed descriptions of the 7 fatal cases of Plasmodium knowlesi malaria and the fatal P. vivax and microscopy-diagnosed P. malariae cases identified in Sabah, Malaysia, 2010–2014. [file 15-1305-Techapp-s1.pdf]

Article DOI: http://dx.doi.org/10.3201/eid2201.151305

Falling *Plasmodium knowlesi* Malaria Death Rates among Adults despite Rising Incidence, Sabah, Malaysia, 2010–2014

Technical Appendix

Fatal Cases of PCR-Confirmed *P. knowlesi* Malaria, 2012–2014

Case 1

A 31 year old man with no known comorbidities presented for the second time in 3 days to a tertiary-referral hospital with seven days of fever, rigors and abdominal pain. On examination he was jaundiced and tachycardic but other vital signs were normal. Respiratory examination was normal. He had tenderness over the epigastric region and right hypochondrium. A provisional diagnosis of ascending cholangitis was made and he was admitted under the surgical team. Blood investigations revealed jaundice (bilirubin 254 µmol/L), acute kidney injury (creatinine 915 µmol/L, urea 37.9 µmol/L) and metabolic acidosis (pH=7.21, bicarbonate 11.5 mmol/L). Blood film was reported as *P. falciparum* with 92,500 parasites/µL. He was commenced on intravenous artesunate and antibiotics (intravenous ceftriaxone and metronidazole and oral doxycycline), however deteriorated rapidly with progressive shock and respiratory distress. He was intubated and ventilated and transferred to the intensive care unit (ICU) where he commenced haemodialysis and inotropic support. Chest radiograph post-intubation was reported as normal. Parasite clearance occurred on day 3 however the patient required ongoing ventilatory and haemodialysis support, with his ICU admission also complicated by *Pseudomonas aeruginosa* ventilator-associated pneumonia for which he was commenced on intravenous ceftazidime. He was extubated and discharged from ICU on day 9, however developed acute respiratory distress the following day while being commenced on haemodialysis. Resuscitation was unsuccessful, and cause of death was reported as acute

pulmonary oedema, although no chest radiograph was performed. Blood cultures taken prior to antibiotics on admission were negative, and PCR confirmed *P. knowlesi* monoinfection.

Case 2

Details of this case have been previously reported (Rajahram et al. Med J Malaysia 2013;68). In brief, a 71 year old woman with a history of hypertension and peptic ulcer disease presented with 5 days of fever associated with chills, rigors, abdominal pain, myalgia and arthralgia. She was jaundiced, tachycardic and tachypnoeic. Bilateral lower zone crepitations were noted on chest auscultation, although her chest radiograph was reported as normal, and arterial blood gas revealed metabolic acidosis. Blood film was reported as *P. malariae* 120,000 parasites/µL and the patient was commenced on intravenous artesunate, oral doxycycline and intravenous ceftriaxone, however her condition deteriorated rapidly requiring intubation and inotropic support. CXR showed bilateral heterogeneous opacities. She died 12 hours later, with a diagnosis of severe malaria complicated by acute kidney injury, hyperbilirubinemia, hypoglycaemia, acute respiratory distress syndrome and metabolic acidosis. Blood cultures done on admission were negative.

Case 3

A 61 year old woman with history of hypertension presented to a district hospital with 7 days of epigastric pain, fever, reduced urine output and dyspnoea. She was tachycardic and tachypnoeic, with an oxygen saturation of 86% on 5 litres of oxygen via facemask. Chest auscultation was unremarkable, and abdominal examination noted hepatomegaly. Blood film was reported as *P. malariae* 320,000 parasites/µL and acute kidney injury was present (creatinine 453 µmol/L, urea 33.8 µmol/L). The patient was commenced on intravenous artesunate and ceftriaxone and transferred to a tertiary referral hospital where she was intubated and ventilated and commenced on inotropic support. Repeat chest auscultation noted bilateral crepitations. Chest radiograph was reported to be normal, and arterial blood gas revealed metabolic acidosis (pH=7.24, bicarbonate 10.3 mmol/L). The patient died 7 hours later, with cause of death reported as acute respiratory distress syndrome. Blood cultures taken on admission prior to antibiotics were negative.

Case 4

A 56 year old man with no known comorbidities was brought unresponsive to a primary health clinic by relatives who reported a one day history of progressive weakness and drowsiness on a background of 3 days of fever, chills, arthralgia and myalgia. On referral to a district hospital he was afebrile, hypotensive (blood pressure 85/60 mmHg) and tachycardic (pulse rate 150 beats per minute). Oxygen saturation was 81% on room air, and 97% on 15 litres oxygen via high flow mask with a respiratory rate of 25 breaths per minute. Glasgow Coma Scale (GCS) was 6/15. Pupils were reactive and measured as 2mm on the right and 4mm on the left. There was no meningism, and neurological examination noted normal tone, symmetrically reduced reflexes and downgoing plantar reflexes. Chest auscultation and abdominal examination were unremarkable. Arterial blood gas revealed severe metabolic acidosis (pH= 7.28, bicarbonate 11.6 mmol/L), and blood film was reported as *P. knowlesi* with 6471 parasites/µL. Chest radiograph was reported as normal. No CT brain or lumbar puncture was performed. The patient was intubated and ventilated, and commenced on inotropic support in addition to intravenous ceftriaxone, artesunate and oral doxycycline. The patient died 41 hours following admission, with blood film prior to death reported as *P. knowlesi* 1134 parasites/µL. Blood cultures taken on admission were negative.

Case 5

A 57 year old woman with a history of type 2 diabetes and hypertension presented to a district hospital with 7 days of fever, lethargy and myalgia. She was febrile and tachycardic, but clinical examination was otherwise unremarkable. Initial blood film was reported as *P. vivax* 9866 parasites/µL and she was commenced on oral chloroquine and primaquine following a single dose of intravenous artesunate. Blood film the following day was reported as *P. knowles*i 20,000 parasites/µL , and the patient was referred to a tertiary referral hospital. Shortly after arrival she was noted to be tachypnoeic (respiratory rate of 34 breaths per minute), and hypoxic (oxygen saturation 70% on 15 liter oxygen via high flow mask), with bilateral crepitations on chest auscultation, and metabolic acidosis on arterial blood gas (pH 7.35, bicarbonate 14.3 mmol/L). She was intubated, ventilated and commenced on intravenous artesunate, ceftriaxone and furosemide, however died shortly after intubation. Cause of death was reported as acute pulmonary oedema, although chest radiograph had not been performed. Blood cultures

performed on admission were negative. Post- mortem re-examination of her initial blood film was reported as *P. knowlesi* 55,111 parasites/µL.

Case 6

A 73 year old man with no known comorbidities presented to a district hospital with a 7-day history of fever, chills and diarrhoea. On examination he was jaundiced and hypotensive, with hepatomegaly. Blood film was reported as *P. malariae* "4+". He was commenced on intravenous artesunate and ceftriaxone and oral doxycycline, however developed worsening respiratory distress, acute kidney injury (creatinine 356 µmol/L) and metabolic acidosis (pH 7.1, bicarbonate 11.8 mmol/L), and was intubated and transferred to the ICU. Chest radiograph showed bilateral lung infiltrates. Tracheal aspirate was positive for acid fast bacilli "2+" and antituberculosis therapy was commenced. Despite haemodialysis and inotropic support, the patient died 3 days later. Cause of death was stated as severe malaria and active tuberculosis with multiorgan failure. Blood cultures performed on admission were negative.

Case 7

A 62 year old woman with a history of hypertension presented with a 6-day history of fever, chills, rigors and dry cough. On examination she was febrile and tachycardic, but examination was otherwise unremarkable and initial chest radiography was normal. Blood film was reported as *P. malariae* with 22,666 parasites/µL, and she was commenced on oral artesunate+mefloquine for uncomplicated malaria. Within 12 hours she became hypotensive (blood pressure 77/49 mmHg) and tachypnoeic (respiratory rate 32 breaths per minute). Oxygen saturation was 98% on room air. Chest auscultation revealed crackles in both lungs and a repeat chest radiograph noted diffuse heterogenous opacities in both lung fields. Arterial blood gas was not available. The patient was intubated and commenced on intravenous artesunate and inotropes, however died within 23 hours of admission. No blood cultures were done. PCR confirmed *P. knowlesi* monoinfection. Later cross-check of initial parasite count showed her true initial *P. knowlesi* parasitemia was 263,772 parasites/µL.

Fatality due to Microscopy-Diagnosed "*P. malariae*"

A previously well 27 year old man presented to a district hospital with a 3-day history of fever and dyspnoea, and reduced conscious state on the day of admission. His Glasgow Coma

Score (GCS) was recorded as 3/15, and pupils were fixed and dilated. Blood pressure was 149/96 mmHg and oxygen saturation was 81% on room air, with bilateral crackles on chest auscultation. Blood film was reported as *P. malariae* “3+”, and kidney injury was present (creatinine 210 µmol/L, urea 9.4 µmol/L). Chest radiograph was reported as normal, and arterial blood gas was not performed. The patient was intubated and ventilated and commenced on intravenous artesunate and antibiotics, however died 21 hours later. No CT brain or lumbar puncture was performed. Cause of death was reported as severe malaria with acute kidney injury and ARDS. Neither blood cultures nor PCR were performed.

Fatal *P. vivax* Malaria

A 53-year-old- man with a history of hypertension presented with 7-day history of fever with chills, rigors, myalgia, non-productive cough and abdominal pain. Heart rate was 125 beats per minute and blood pressure was 159/34 mmHg, with examination otherwise normal. Blood investigations noted thrombocytopenia (platelets 74,000 cells/µL) and mildly elevated bilirubin (21.5 µmol/L), but other blood parameters on admission were normal, including haemoglobin (17 g/dL), creatinine (108 µmol/L) and urea (6 µmol/L). Blood film was reported as *P.vivax* with 2090 parasites/µL, and the patient was commenced on oral chloroquine and primaquine. The following day the parasite count was 890 parasites/µL, however the patient became hypotensive (blood pressure 95/51 mmg Hg), tachypnoeic (respiratory rate 34 breaths/minute) and hypoxic (oxygen saturation 70% on room air) with generalized crackles on chest auscultation, and he was intubated and transferred to ICU. Chest radiograph showed bilateral heterogeneous opacities, and FiO2:Pa02 ratio was 132. The patient was commenced on intravenous artesunate and intravenous antibiotics in addition to inotropic support, and subsequently required haemodialysis for acute kidney injury (creatinine 316 µmol/L, urea 19.6 µmol/L). Despite supportive care the patient died 4 days after admission. Cause of death was reported as severe malaria with ARDS and acute kidney injury. PCR identified *P. vivax* monoinfection. Blood cultures done on admission were negative.

Technical Appendix Table 1. Demographic, clinical and laboratory features of fatal cases of PCR-confirmed *P. knowlesi*, and one microscopy-diagnosed "*P. malariae*"

					Severity criteria on presentation								
No.	Sex, age, y	Nationality	Initial microscopic diagnosis, parasites/µL	PCR	Acute Kidney Injury (Cr >265 µmol/L)	Jaundice (Bil >43 µmol/L) with Cr >132 µmol/L or parasite count >20,000 parasites/µL	Hypotension (BP ≤80 mm Hg)	Metabolic acidosis (HCO_3 <15 mmol/L)	Respiratory distress (SaO_2 <94%)*	Coma	Time to death, h	Initial antimalarial treatment	Complications developing following commencement of antimalarial treatment
1	M, 31	Malaysian	Pf 92,500	Pk	Yes (Cr 915 µmol/L)	Yes (Bil 254 µmol/L)	No	Yes (HCO_3 11.5 mmol/L)	No	No	117	iv artesunate, oral doxycycline	Shock, respiratory distress
2	F, 71	Malaysian	Pm 120,000	Pk	Yes (Cr 662 µmol/L)	Yes (Bil 108 µmol/L)	No	Yes (HCO_3 4.2 mmol/L)	No	No	12	iv artesunate, oral doxycycline	Shock, respiratory distress (ARDS)
3	F, 61	Malaysian	Pm 320,000	Pk	Yes (Cr 453 µmol/L)	Yes (Bil 315 µmol/L)	No	Yes (HCO_3 10.3 mmol/L)	Yes (SaO2 88%)	No	5	iv artesunate	Shock
4	M, 56	Filipino	Pk 6471	Pk	No	No	No	Yes (HCO_3 11.6 mmol/L)	Yes (SaO_2 81%)	Yes	41	iv artesunate, oral doxycycline	Shock
5	F, 57	Filipino	Pv 9866[1]	Pk	No	Yes (Bil 46 µmol/L, Cr 143 µmol/L)	No	N/A	No	No	63	iv artesunate (single dose) followed by CQ and PQ	Shock, respiratory distress (ARDS), metabolic acidosis
6	M, 73	Malaysian	Pm "4+"[2]	Pk	No	Yes (Bil 234 µmol/L)[4]	Yes (BP 80/49 mm Hg)	No	No	No	71	iv artesunate, oral doxycycline	Respiratory distress, AKI, metabolic acidosis
7	F. 62	Malaysian	Pm 22,666[3]	Pk	No	N/A	No	N/A	No	No	23	oral artesunate/ mefloquine	Shock, respiratory distress
X	M, 27	Filipino	Pm "3+"[2]	N/A	No	N/A (Cr 210 µmol/L)	No	N/A	Yes (SaO_2 81%)	Yes	21	iv artesunate	shock

Pk = P. knowlesi; Pv = P. vivax, Pf = P. falciparum; Pm = P. malariae; Cr = creatinine; Bil = bilirubin, iv = intravenous; BP = blood pressure; HCO_3 = bicarbonate; SaO_2 = oxygen saturation; CQ = chloroquine; PQ = primaquine; ARDS = acute respiratory distress syndrome; AKI = acute kidney injury; N/A = not available.

[1]Post-mortem re-examination of blood film reported as *P. knowlesi* 55,111 parasites/µL.

[2] "3+" refers to 1 - 10 parasites per thick field, and "4+" refers to >10 parasites per thick field.

[3]Post-mortem re-examination of blood film reported as *P. knowlesi* 262,772 parasites/µL.

[4]Creatinine 132 µmol/L and exact parasite count recorded only as "4+."

Note: Blood culture taken before antibiotics were negative in all patients except case 7, where they were not performed. Detailed case descriptions are listed in the text above.

Technical Appendix Table 2. Demographic, clinical and laboratory features of reported PCR-confirmed *P. knowlesi* deaths

Details	Case 1	Case 2	Case 3	Case 4	Case 5	Case 6	Case 7
Age	31	71	61	56	57	73	62
Sex	Male	Female	Female	Male	Female	Male	Female
Nationality	Malaysian	Malaysian	Malaysian	Filipino	Filipino	Malaysian	Malaysian
Time to death, hours	117	12	5	41	63	71	23
Blood pressure, mmHg	129/72	127/87	112/79	85/60	193/96	80/49	116/71
Heart rate per minute	108	111	124	150	129	72	111
Respiratory Rate per minute	24	38	26	25	22	20	22
Oxygen saturation on room air	97	96	88	81	98	100	96
PaO2:FiO2 ratio	297	86	159	164	79 [1]	288	NA
Axillary temperature, °C	37.4	37.0	36.8	36.2	39.0	37.7	39.9

Details	Case 1	Case 2	Case 3	Case 4	Case 5	Case 6	Case 7
Hemoglobin, g/dL (females 12.0-16.0, males 13.5-17.5)	12.9	14.1	12.6	13.8	11.1	11.0	12.0
WBC count, x 10^3 cells/mL (4.5-11)	6.9	13.3	20.5	12.1	5.3	8.2	11.2
Platelet count, x 10^3 cells/µL (150-450)	25	53	8	73	26	67	60
Serum creatinine, µmol/L (63-133)	915	662	453	171	143	132	124
Serum urea, µmol/L (1.0–8.3)	37.9	36.3	33.8	11.8	12.1	13.8	6.1
Total serum bilirubin, µmol/L (<17)	254	108	315	43	46	234	NA
Serum aspartate aminotransferase, U/L (<37)	67	322	732	373	NA	61	NA
Serum alanine aminotransferase concentration, U/L (<40)	NA	145	258	143	NA	36	NA
Serum albumin, g/L (35-60)	28	24	20	24	27	20	NA
Serum bicarbonate, mmol/L (18-23)	11.5	4.2	10.3	11.6	14.3	16	NA
Serum lactate mmol/L (0.5-2.2)	3.6	NA	NA	NA	NA	NA	NA
Blood cultures	Negative	Negative	Negative	Negative	Negative	Negative	Not done
Initial microscopic diagnosis, parasites/µL	*P. falciparum* 92,500	*P. malariae* 120,000	*P. malariae* 320,000	*P. knowlesi* 6471	*P. vivax* 9866[2]	*P. malariae* "4+"	*P. malariae* 22,666[3]
PCR result	*P. knowlesi*	*P. knowlesi*	*P. knowlesi*	*P.knowlesi*	*P. knowlesi*	*P. knowlesi*	*P.knowlesi*
Initial anti-malarial therapy received	IV artesunate/oral doxycyline	IV artesunate/oral doxycycline	IV artesunate	IV artesunate/oral doxycycline	IV artesunate (single dose), followed by oral chloroquine and primaquine	IV artesunate/oral doxycycline	oral artesunate + melfoquine

NOTE. Laboratory reference ranges are given in parentheses. NA, not available; IV, intravenous
Unless otherwise stated, clinical and laboratory parameters are those obtained on admission.
